# Dynamics of a brake system governed by modified Burridge-Knopoff-Pad model

**DOI:** 10.1016/j.heliyon.2025.e41999

**Published:** 2025-01-23

**Authors:** Oma Nfor Nkeh, Akoni Brikly Njinabo, Waindim Yisa Tufoin Albert, Kenfack Djifack Hunnel

**Affiliations:** a Department of Physics, Higher Teacher Training College Bambili, The University of Bamenda, P. O. Box 39, Bambili, Cameroon; b Department of Physics, Faculty of Science Bambili, The University of Bamenda, P. O. Box 39, Bambili, Cameroon; c Department of Mechanical Engineering, Higher Technical Teacher Training College Bambili, The University of Bamenda, P. O. Box 39, Bambili, Cameroon

**Keywords:** Automobile brake system, Burridge-Knopoff-Pad model, Brake squeal noise, Nonlinear vibrations, Linear stability analysis

## Abstract

Despite widespread investigations to improve on the performance of automobile brake systems, the undesirable phenomenon of disc brake squeal noise remains a very challenging problem to solve. Consequently, we thus propose a basic theoretical brake system based on the modified Burridge-Knopoff-Pad model in this study; in order to elucidate on brake squeal noise and other nonlinear vibrations. Linear stability analysis clearly depicts the stability/instability of some spatial-homogeneous steady states, when some parameters of the system are varied. Our investigation brings into sharp focus the role of nonlinearity, and strongly suggests that the instability of perturbed continuous wave is physically manifested as brake squeal noise. Result of numerical simulations clearly underscores the displacement and velocity profiles of the block-pad brake dynamical system.

## 1 Introduction

Most automobile systems need to be properly designed in order to adequately understand and model the nature of frictional forces involved during the motion of two or more surfaces in contact. Excessive frictional force between moving objects generally results in limit cycles, triggered by stick-slip instability [1], [2]. A brake system is generally a mechanical setup which inhibits motion, and can be categorized as mechanical, hydraulic or power. Friction-induced vibrations are ubiquitous in cutting machine [3], artificial joints [4] and elastic disc [5]. Furthermore, the undesirable phenomenon of brake squeal noise is inextricably linked to friction-induced vibrations in automobile braking systems [6]. Extensive experimental, numerical and analytical investigations have been carried out in recent years; in order to predict and prevent the occurrence of brake squeal noise [7], [8].

The study of brake dynamics is quite crucial in guaranteeing the safety and performance of a plethora of automobile systems. It is important to reiterate that frictional force determines the efficiency of vibrating mechanical systems and typically serves as a stabilizing agent because it saps out energy from the system [9]. The Burridge-Knopoff model of earthquake has been recently considered as the basic model to describe the interface between the brake disc and pad [10]. The pad is generally viewed as a one degree-of-freedom system that is attached to a spring and a damper; which are in turn connected to an upper fixed surface called the caliper. The combined Burridge-Knopoff and pad models will be henceforth referred to as Burridge-Knopoff-Pad model [10], in this scientific investigation. From a practical standpoint, the disc brake system is made up of a disc that is attached to the axle hub; hence rotating with the wheel. When the brake is effectively applied, the pad which is made of friction material is pressed against the rotating wheel. The stationary mounted caliper generally contains hydraulic pistons which press the pad onto the disc with high pressure upon application of the brakes. The rotating disc generally slows down as a result of the frictional force between the pad and disc that continuously transforms kinetic energy to heat, which is discharged by appropriate cooling process [11]. A small part of the rotational kinetic energy is equally transformed to noise.

Different possible mechanisms have been brought forth to elucidate on the undesirable disc brake vibrations induced by frictional forces [12]. The stick-slip phenomenon underscores a situation where a block is stuck to the moving surface, because it is being held in place by frictional forces. However, the block can slip and eventually exhibit damped oscillatory motion when the spring force supersedes the static friction. Stick-slip motion is generally observed when static friction exceeds dynamic friction [13]. It has been further established that the frequency of stick-slip motion is proportional to the disc speed, with a critical disc speed value derived; beyond which stick-slip motion is impossible [14]. The theory of sprag-slip motion as a possible mechanism of friction-induced disc brake vibration was equally brought into sharp focus [15]. In this sprag-slip model, the pad is viewed as a rod pivoted around a fixed point with the braking force pushing the other end of the rod onto the rough surface. The coupling of two vibrational modes of brake components like caliper, pad, disc rotor etc, has the potential of triggering brake squeal noise [16]. The mode of application of brake force can equally lead to squealing. This become pronounce in automobile systems that are characterized by velocity-weakening friction law, as underscored by Kinkaid et al. [11]. The mechanisms highlighted above are quite limited because it can only predict the initiation of squeal noise in automobile braking systems. In fact, Kinkaid et al. elucidated on the complex issue of brake squeal from experimental, computational and analytical perspectives [11]. Their comprehensive review incorporated experimental investigations which brought into sharp focus the following pertinent issues: (i) The squealing of brake system can occur at distinct frequencies. (ii) Low amplitude of vibrations during squealing. (iii) The natural frequency of braking system is slightly higher than their squealing frequencies. (iv) Friction-induced instability generally triggers squealing. The magnitude of the friction is directly proportional to the propensity of brake system to squealing. In the conclusion of their work, Kinkaid et al. pointed out that most studies on brake mechanisms were limited to linear systems which is insufficient in predicting the evolution of the system beyond instability. Consequently, the aim of this study is to consider a basic theoretical nonlinear mechanical model of brake system; in order to further elucidate on the squeal phenomena.

Considering the limitations inherent in the classical methods of predicting brake performance, there is urgent need to incorporate novel approach like the Burridge-Knopoff-Pad model [10]. It is important to stress that the original Burridge-Knopoff model is very reliable in mimicking real seismic activities that obey the Gutenberg-Richter law [17], [18], [19], [20], [21]. In the Burridge-Knopoff model of earthquakes, the interaction of two tectonic plates that characterize a geological fault is modeled as elastically coupled chain of blocks; in which one of the plates is subjected to frictional force of the surface of the other plate [17], [18]. Due to the reliability of the Burridge-Knopoff model, the Burridge-Knopoff-Pad model was thus established as a novel approach to brake dynamics [10]. However from a practical standpoint, there are still limitations with the Burridge-Knopoff-Pad model because not all components of the automobile suspension system are incorporated. Furthermore, there are variations in the effective brake force along the pad in the axis of disc rotation. This is as a result of variation in frictional force that triggers an angular moment around the centroid of the pad. The present study thus seeks to address some of the shortcomings highlighted by proposing a modified Burridge-Knopoff-Pad model, in which nonlinear springs are incorporated [22], [23], [24], [25], [26], [27], [28]. This becomes more pertinent, considering the limited scientific investigations on the influence of nonlinearity on brake squeal noise [29]. With such a robust braking system governed by the modified Burridge-Knopoff-Pad model, only the application of very strong braking force can perturb the system.

In order to fully achieve the goal of our scientific investigation, this work will be structured in the following way. We present the modified Burridge-Knopoff-Pad model of brake system in **section** 2. The model equations are further refined using appropriate dimensionless parameters and the continuum limit approximation. In **section** 3, we carry out a comprehensive linear stability analysis of the system to determine which parameter values influence brake squeal noise. Numerical simulations are performed in **section** 4. This is to establish various nonlinear vibrational profiles of the position and speed of the block-pad brake system. Finally, **section** 5 gives a discussion and conclusion on the work by highlighting the important results obtained.

## 2 The brake model equations

**Figure 1:**
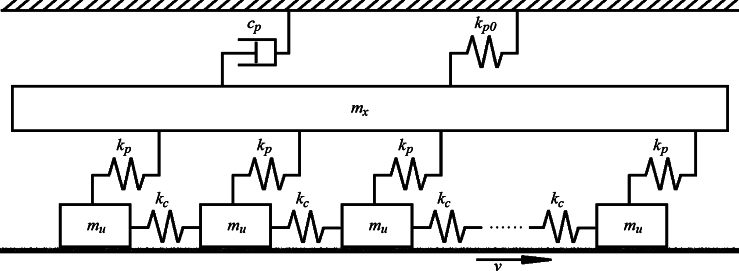
Mechanical interpretation of block-spring configuration mimicking the modified Burridge-Knopoff-Pad model of brake system. The blocks of equal masses *m*_*u*_ that rest on rough rotor disc surface (rotating with constant speed *v*), are linearly connected to nearest neighbors by springs of strength *k*_*c*_, and to the pad of mass *m*_*x*_ by nonlinear springs of strength *k*_*p*_ = *k*_*p*_(*Y*_*n*_). The pad is in turn coupled to the stationary caliper by a damper *c*_*p*_, and a linear spring of constant *k*_*p*0_. This mechanical set up is adapted from reference [10]. It should be noted that there are two parallel vibrating coordinate systems given by *X* and *Y*_*n*_ (unlike the Cartesian coordinate system in which *X* and *Y* are generally perceived to be mutually perpendicular to each other). Concretely, *X* is the displacement of the pad of mass *m*_*x*_; while the motion of the block masses *m*_*u*_ that rest on the rotating disc surface is given by *Y*_*n*_.

Fig. 1 depicts a brake system governed by the modified Burridge-Knopoff-Pad model. Each block of mass $m_{u}$, is connected by linear springs to their nearest neighbors with spring constant $k_{c}$. The blocks are also connected to a pad of mass $m_{x}$, by nonlinear springs of strength $k_{p}=k_{p}(Y_{n})$. The pad is in turn coupled to the stationary caliper by a damper of damping coefficient $c_{p}$ and a linear spring of constant $k_{p0}$. The blocks rest on the rough disc surface that rotates with constant speed *v*. The blocks are primarily considered as the points of contact between the two plates of the brake system, i.e. the stationary caliper and disc rotor. Fig. 1 clearly captures a basic theoretical nonlinear model, with all the important components that characterize a brake system. The model is quite simple compared to the complex brake systems highlighted in various experimental studies [11] (check the references therein). We have deliberately considered a simple nonlinear brake system in Fig. 1 because of our theoretical background. This study further underscores the influence of nonlinearity on brake dynamics, by theoretically advancing our knowledge on the elusive problem of brake squeal and other nonlinear vibrations. We hope that mechanical engineers will be inspired by our basic nonlinear brake model, in order to build a more practical robust brake system.

The equations of motion of the brake system that is governed by the modified Burridge-Knopoff-Pad model reads

(1a) $$m_{u}{\overset{¨}{Y}}_{n}=k_{c}(Y_{n+1}-2Y_{n}+Y_{n-1})-k_{p}(Y_{n}-X)-F(v+{\overset{˙}{Y}}_{n}),$$

(1b) $$m_{x}\overset{¨}{X}=-c_{p}\overset{˙}{X}+k_{p}\sum_{n}(Y_{n}-X)-k_{p0}X.$$

We have that

(2) $$k_{p}=k_{p}(Y_{n})=k_{0}[1+\frac{k_{non}}{k_{0}}Y_{n}^{2}],$$

is the nonlinear spring strength that is dependent on $n^{th}$ block position $Y_{n}$ [22], [23], [24], [25], [26], while *X* is the position of the pad. In fact, $k_{p}$ is the effective coupling coefficient between the blocks (each of mass $m_{u}$) and pad. Nonlinear springs are generally classified as hardening/softening, depending on whether their strengths gradually increase/decrease respectively; when progressively subjected to intensive compression or extension [27], [28]. The stiffness of the brake pad lining material was equally shown to be governed by relation (2), for a two degree of freedom brake-disc system [30]. It should be noted that when all velocity components of the system vanish and all springs are relaxed, then the pad and each block are considered to be at their rest positions. In the limiting case of nonlinear spring coefficient i.e. $k_{non}\to 0$ in Eq. (2), we have that $k_{p}=k_{p}(Y_{n})\approx k_{0}$; and the system of Eq. (1a), (1b) reduces to the Burridge-Knopoff-Pad model [10]. As shown in Fig. 1, the block masses $m_{u}$ are the only points of contact between the brake system and rotating disc surface. By considering that the unit normal between the pad and disc is $\overset{ˆ}{z}$, it is therefore clear that the normal coordinate system *Z* is perpendicular to both *X* and *Y* coordinates. This is because the *X* and *Y* coordinate systems are considered parallel to each other in our current investigation.

In the absence of the pad, damper and linear spring of constant $k_{p0}$; the system of Eq. (1a), (1b) degenerates to the equation of motion that governs the modified Burridge-Knopoff model of earthquake fault [22]. It is quite instructive to reiterate that our current investigation is within a peculiar mechanical system, that is quite innovative in disc brake automobile modeling.

The form of the frictional law *F*, of interest in this study is given by

(3) $$F(\overset{˙}{Y})=F_{0}\phi (\overset{˙}{Y}/v_{1}),$$

known as the velocity-dependent friction force with $v_{1}$ being a characteristic velocity [19]. The frictional force that opposes the relative motion between the black masses and disc is given by Eq. (3). Even though this frictional force acts parallel to the *X* or *Y* coordinate system, it is important to highlight that it is triggered as a result of the normal reaction force that exists between the masses $m_{u}$ and the rotating disc surface [11]. Hence, it is now becoming increasingly clear that the normal force that exists between the pad and disc is incorporated in the frictional force law (3). From the analysis in reference [22], it is now necessary for us to introduce the following parameters: $T=\omega_{p}t,\;\;\omega_{p}^{2}=k_{0}/m_{u},\;\;D_{0}=F_{0}/k_{0},\;U_{n}=(k_{0}/F_{0})Y_{n}$ and $V=(k_{p0}/c_{p})X$. For spatially homogeneous system of N blocks, Eq. (1a), (1b) can now be re-written as

(4a) $${\overset{¨}{U}}_{n}=l^{2}(U_{n+1}-2U_{n}+U_{n-1})-(U_{n}-\gamma_{2}V)(1+\gamma_{1}U_{n}^{2})-\phi (2\alpha \nu +2\alpha {\overset{˙}{U}}_{n}),$$

(4b) $$\overset{¨}{V}=-\gamma_{3}\overset{˙}{V}+(\gamma_{4}U_{n}-\gamma_{5}V)(1+\gamma_{1}U_{n}^{2})-\gamma_{6}V,$$

where

$$l^{2}=k_{c}/k_{0},\;\nu =v/(\omega_{p}D_{0}),\;2\alpha =\omega_{p}D_{0}/v_{1},\;\gamma_{1}=k_{non}D_{0}^{2}/k_{0},$$

$$\gamma_{2}=c_{p}/D_{0}k_{p0},\;\gamma_{3}=\sqrt{m_{u}}c_{p}/\sqrt{k_{0}}m_{x},\;\gamma_{4}=ND_{0}m_{u}k_{p0}/m_{x}c_{p},$$

(5) $$\gamma_{5}=Nm_{u}/m_{x},\;\gamma_{6}=m_{u}k_{p0}/m_{x}k_{0}.$$

Equation (4a), (4b) is a set of two coupled dissipative nonlinear ordinary differential equations of motion, with variable $U_{n}$ in the discrete regime while *V* is the continuum variable. It is important to stress that Eq. (4a), (4b) can not be easily solved analytically because of the undefined form of the frictional force law *ϕ*, the nonlinearity introduced by the $\gamma_{1}$ term and the discreteness. However if the displacement $U_{n}$ varies slowly from one mass to the other (i.e. $k_{c}>>k_{0}$), we can then implore the continuum limit approximation

(6) $$U_{n}(T)\to U(x,T),\;\;x=na,\;\;U_{n\pm 1}=U\pm a\frac{\partial U}{\partial x}+\frac{a^{2}}{2}\frac{\partial^{2}U}{\partial x^{2}}+....,$$

where *a* is the lattice spacing. Consequently, Eq. (4a), (4b) is then transformed to

(7a) $$\frac{\partial^{2}U}{\partial T^{2}}=a^{2}l^{2}\frac{\partial^{2}U}{\partial x^{2}}-(U-\gamma_{2}V)(1+\gamma_{1}U^{2})-\phi (2\alpha \nu +\alpha \overset{˙}{U}),$$

(7b) $$\frac{\partial^{2}V}{\partial T^{2}}=-\gamma_{3}\frac{\partial V}{\partial T}+(\gamma_{4}U-\gamma_{5}V)(1+\gamma_{1}U^{2})-\gamma_{6}V.$$

It is important to recall that Carlson and Langer proposed the initial form of the frictional force law given by [19]

(8) $$\phi (x)=\frac{1}{1+|x|}sgn(x).$$

The frictional force law ϕ(x) given by Eq. (8) is inextricably linked to Eq. (3), because *X* and *Y* are parallel coordinate systems. Equation (8) thus gives a generic form of the friction force law, which can still be re-written in *Y*-coordinate system as sgn(y)/(1+|y|). A careful examination of Eq. (8) reveals that for zero velocity (i.e. ϕ(0)), the frictional force adopts any numerical value within ±1; regardless of the force required to oppose that of the springs. However, in order for the law to be very realistic by incorporating the transition between localized and greater delocalized vibrations; Carlson et al. subsequently modified the frictional law (8) to now read [20]

(9) $$\phi (x)=\left\{ \begin{array}{c} (-\infty ,1],\;\;x=0\\ \frac{1-\sigma }{1+\frac{x}{1-\sigma }},\;\;x>o.\; \end{array}\right.$$

It is now becoming increasingly clear that for zero velocity, the modified friction force law (9) is constrained within the interval (−∞,1]. The braking system no longer sustains negative velocity with the value of the maximum sliding friction force fixed at 1−σ; where *σ* is the acceleration of a block at the instant when slipping begins [22]. On the other hand, the corresponding maximum value of the static frictional force is maintained at unity. We are interested in the dynamic frictional force of the brake system by considering the linear approximation of the function ϕ(x), in order for Eq. (7a), (7b) to be re-written as [24], [26]

(10a) $$\frac{\partial^{2}U}{\partial T^{2}}=a^{2}l^{2}\frac{\partial^{2}U}{\partial x^{2}}-(U-\gamma_{2}V)(1+\gamma_{1}U^{2})+2\alpha \frac{\partial U}{\partial T},$$

(10b) $$\frac{\partial^{2}V}{\partial T^{2}}=-\gamma_{3}\frac{\partial V}{\partial T}+(\gamma_{4}U-\gamma_{5}V)(1+\gamma_{1}U^{2})-\gamma_{6}V.$$

The last term in Eq. (10a), (10b) with *α*-coefficient, is thus the main parameter that characterizes the frictional force. It is important to note that for a special case of $\gamma_{1}=0\;(i.e.\;k_{non}=0)$, the coupled nonlinear Eqs. (10a), (10b) reduces to a linear system of coupled partial differential equations which obey the superposition principle [28].

## 3 Linear stability analysis

Since brake squeal noise is generally triggered as a result of instability of the brake mechanism, it is thus incumbent for us to scrupulously carry out a linear stability analysis of system (10a), (10b). This will give us the opportunity to characterize the stability/instability of the homogeneous states of Eq. (10a), (10b), hence devising appropriate means of predicting and controlling brake squeal noise. By solving the following simultaneous equations

(11a) $$(U-\gamma_{2}V)(1+\gamma_{1}U^{2})=0,$$

(11b) $$(\gamma_{4}U-\gamma_{5}V)(1+\gamma_{1}U^{2})-\gamma_{6}V=0,$$

it is possible to easily obtain the solutions. Hence, the three spatially-homogeneous steady states of Eq. (10a), (10b) are given by

(12a) $$(U_{1}^{⁎},V_{1}^{⁎})=(0,\;0),$$

(12b) $$(U_{2}^{⁎},V_{2}^{⁎})=(\sqrt{\frac{\gamma_{6}-(\gamma_{4}\gamma_{2}-\gamma_{5})}{\gamma_{1}(\gamma_{4}\gamma_{2}-\gamma_{5})}},\;\sqrt{\frac{\gamma_{6}-(\gamma_{4}\gamma_{2}-\gamma_{5})}{\gamma_{1}\gamma_{2}^{2}(\gamma_{4}\gamma_{2}-\gamma_{5})}}),$$

(12c) $$(U_{3}^{⁎},V_{3}^{⁎})=(-U_{2}^{⁎},\;-V_{2}^{⁎}).$$

From a practical standpoint, we will incorporate hardening nonlinear springs in the coupling of the block of masses to the pad. This means that the strength of the nonlinear spring increases gradually when subjected to increase extension/compression, i.e. $\gamma_{1}>0$. For real values of spatially-homogeneous steady states, the condition $\gamma_{6}>\gamma_{4}\gamma_{2}-\gamma_{5}>0$ must be imposed.

We will now carry out a linear stability analysis in order to effectively determine the stability of the steady states $\left(U^{⁎},\;V^{⁎}\right)$ given in Eq. (12a), (12b), (12c). Consequently, we linearize Eq. (10a), (10b) by searching for solution in the form [31]

(13a) $$U(x,T)=U^{⁎}+\Delta U(x,T),$$

(13b) $$V(x,T)=V^{⁎}+\Delta V(x,T).$$

The amplitudes of the perturbations to U(x,T) and V(x,T), are respectively given by ΔU(x,T) and ΔV(x,T). Upon substitution of Eq. (13a), (13b) into Eq. (10a), (10b) and linearizing, we obtain

(14a) $$\frac{\partial^{2}\Delta U}{\partial T^{2}}=a^{2}l^{2}\frac{\partial^{2}\Delta U}{\partial x^{2}}+2\alpha \frac{\partial \Delta U}{\partial T}-(1+3\gamma_{1}U^{⁎2}-2\gamma_{1}\gamma_{2}U^{⁎}V^{⁎})\Delta U+(\gamma_{2}+\gamma_{1}\gamma_{2}U^{⁎2})\Delta V,$$

(14b) $$\frac{\partial^{2}\Delta V}{\partial T^{2}}=-\gamma_{3}\frac{\partial \Delta V}{\partial T}+(\gamma_{4}+3\gamma_{1}\gamma_{4}U^{⁎2}-2\gamma_{1}\gamma_{5}U^{⁎}V^{⁎})\Delta U-(\gamma_{5}+\gamma_{6}+\gamma_{1}\gamma_{5}U^{⁎2})\Delta V.$$

Without loss of generality we set $a^{2}l^{2}=1$, and assume the perturbations ΔU(x,T) and ΔV(x,T) to be [31]

(15a) $$\Delta U(x,T)=U_{0}\;exp(\lambda T+ikx),$$

(15b) $$\Delta V(x,T)=V_{0}\;exp(\lambda T+ikx).$$

$U_{0}$ and $V_{0}$ in Eq. (15a), (15b) are constants, *λ* is the growth factor, while *k* is the real wavenumber. Upon substituting Eq. (15a), (15b) into Eq. (14a), (14b) and simplifying, yields the system of equations represented in the matrix form

(16) $$\left(\begin{array}{c} m_{11}\;\;\;\;\;\;\;\;\;\;\;\;m_{12}\\ m_{21}\;\;\;\;\;\;\;\;\;\;\;\;m_{22} \end{array}\right)\left(\begin{array}{c} U_{0}\\ V_{0} \end{array}\right)=\left(\begin{array}{c} 0\\ 0 \end{array}\right),$$

with all the coefficients $m_{ij}$ given by: $m_{11}=1+\lambda^{2}+k^{2}-2\alpha \lambda +3\gamma_{1}U^{⁎2}-2\gamma_{1}\gamma_{2}U^{⁎}V^{⁎},\;m_{12}=-(\gamma_{2}+\gamma_{1}\gamma_{2}U^{⁎2}),\;m_{21}=-(\gamma_{4}+3\gamma_{1}\gamma_{4}U^{⁎2}-2\gamma_{1}\gamma_{5}U^{⁎}V^{⁎}),\;m_{22}=\gamma_{5}+\gamma_{6}+\lambda^{2}+\gamma_{3}\lambda +\gamma_{1}\gamma_{5}U^{⁎2}$. It should be noted that the matrix system of Eq. (16) can only admit non-trivial solutions when the determinant of the 2x2 matrix vanishes. Consequently, this naturally lead us to the following characteristic equation

(17) $$\lambda^{4}+p_{3}\lambda^{3}+p_{2}\lambda^{2}+p_{1}\lambda +p_{0}=0,$$

where: $p_{3}=\gamma_{3}-2\alpha ,\;p_{2}=1+k^{2}+\gamma_{5}+\gamma_{6}-2\alpha \gamma_{3}+\gamma_{1}(3+\gamma_{5})U^{⁎2}-2\gamma_{1}\gamma_{2}U^{⁎}V^{⁎},\;p_{1}=\gamma_{3}(1+k^{2}+3\gamma_{1}U^{⁎2}-2\gamma_{1}\gamma_{2}U^{⁎}V^{⁎})-2\alpha (\gamma_{5}+\gamma_{6}+\gamma_{1}\gamma_{5}U^{⁎2}),\;p_{0}=(\gamma_{5}+\gamma_{6}+\gamma_{1}\gamma_{5}U^{⁎2})(1+k^{2}+3\gamma_{1}U^{⁎2}-2\gamma_{1}\gamma_{2}U^{⁎}V^{⁎})-\gamma_{2}(1+\gamma_{1}U^{⁎2})(\gamma_{4}+3\gamma_{1}\gamma_{4}U^{⁎2}-2\gamma_{1}\gamma_{5}U^{⁎}V^{⁎})$.

It is thus clear that the coefficients $p_{2},\;p_{1},\;p_{0}$, are all functions of the wavenumber *k*, with the solution of Eq. (17) given by [31]

(18a) $$\lambda_{1,2}=-\frac{p_{3}}{4}-\frac{1}{2}\sqrt{\frac{p_{3}^{2}}{4}-\frac{2p_{2}}{3}+\Omega }\pm \frac{1}{2}\sqrt{\frac{p_{3}^{2}}{2}-\frac{4p_{2}}{3}-\Omega +\frac{p_{3}^{2}-4p_{2}p_{3}+8p_{0}}{4\sqrt{\frac{p_{3}^{2}}{4}-\frac{2p_{2}}{3}+\Omega }}},$$

(18b) $$\lambda_{3,4}=-\frac{p_{3}}{4}+\frac{1}{2}\sqrt{\frac{p_{3}^{2}}{4}-\frac{2p_{2}}{3}+\Omega }\pm \frac{1}{2}\sqrt{\frac{p_{3}^{2}}{2}-\frac{4p_{2}}{3}-\Omega -\frac{p_{3}^{2}-4p_{2}p_{3}+8p_{0}}{4\sqrt{\frac{p_{3}^{2}}{4}-\frac{2p_{2}}{3}+\Omega }}}.$$

We have that

(19a) $$\Omega =\frac{1}{3}\{\sqrt[{3}]{\frac{\Delta_{2}+\sqrt{\Delta_{2}^{2}-4\Delta_{1}^{3}}}{2}}+\frac{\Delta_{1}}{\sqrt[{3}]{\frac{\Delta_{2}+\sqrt{\Delta_{2}^{2}-4\Delta_{1}^{3}}}{2}}}\},$$

(19b) $$\Delta_{1}=p_{2}^{2}-3p_{1}p_{3}+12p_{0},$$

(19c) $$\Delta_{2}=2p_{2}^{2}-9p_{1}p_{2}p_{3}+27p_{0}p_{3}^{2}+27p_{1}^{2}-72p_{0}p_{2}.$$

By considering the parameter values of our brake system, the roots of Eq. (17) may either be a positive real number, negative real number, or complex number. It is important to note that the nature of these roots are very sensitive parameters used to characterize the stability/instability of the perturbed homogeneous states. For instance when the roots are all positive real numbers, it is a clear indication that the continuous waves are unstable; with high propensity of triggering brake squeal noise [7], [32]. On the other hand, for negative real values of $\lambda_{i}\;(i=1,\;2,\;3,\;4)$ for any wavenumber *k*; it physically signifies linear stability of the homogeneous states $\left(U^{⁎},\;V^{⁎}\right)$. Hence, every perturbed signal likely to generate brake squeal noise will vanish in the shortest possible time. For complex values of $\lambda_{i}$, the imaginary part generally captures time harmonic oscillations of the perturbations whereas positive real part signifies exponential amplification; otherwise damping for negative real part. Sometimes with a positive real part, a very interesting phenomenon may occur in which the continuous wave grow in magnitude and forces the system from the homogeneous state to any configuration of defined wavenumber *k* [31].

**Figure 2:**
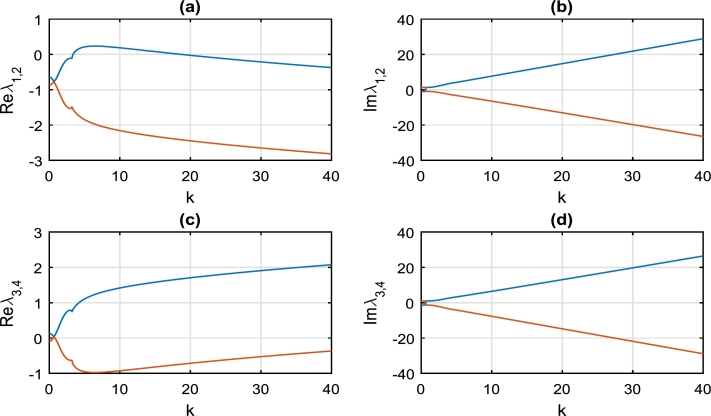
Variation of growth rate *λ* as a function of wavenumber *k*, dictated by solution (18a), (18b). This is evaluated at spatially-homogeneous state $\left(U_{1}^{⁎},\;V_{1}^{⁎})=(0,\;0\right)$, with parameter values given by: *α* = 0.01, *γ*_1_ = 1.00, *γ*_2_ = 1.00, *γ*_3_ = 1.50, *γ*_4_ = 0.75, *γ*_5_ = 0.50, *γ*_6_ = 1.25. (a) The blue curve is for *Reλ*_1_, while the red curve is for *Reλ*_2_. (b) The blue curve is for *Imλ*_1_, while the red curve is for *Imλ*_2_. (c) The blue curve is for *Reλ*_3_, while the red curve is for *Reλ*_4_. (d) The blue curve is for *Imλ*_3_, while the red curve is for *Imλ*_4_.

Fig. 2 clearly depicts the evolution of real and imaginary parts of the eigenvalues $\lambda_{i}$ as a function of wavenumber *k*, evaluated at spatial-homogeneous state $\left(U_{1}^{⁎},\;V_{1}^{⁎})=(0,\;0\right)$. It is very obvious from Fig. 2(a,c) that values of $Re\lambda_{2}$ and $Re\lambda_{4}$ are all negative in the range of k∈[0,40], hence cannot be exploited to effectively characterize the instability of the spatial-homogeneous state $\left(U_{1}^{⁎},\;V_{1}^{⁎})=(0,\;0\right)$. However we notice that $Re\lambda_{1}$ is positive for k∈[4,20], while $Re\lambda_{3}$ is positive for all values of *k*. Perturbations around the homogeneous state (0,0) are always unstable for any wavenumber *k*, hence the brake system will always experience the phenomenon of brake squeal noise. Around the wavenumber k=1.00, a careful observation of Fig. 2(b,d) reveals that $Im\lambda_{1,2}=Im\lambda_{3,4}=0$; while $Re\lambda_{1,2}<0$ and $Re\lambda_{3,4}\approx 0$ in Fig. 2(a,c). This is a clear indication that perturbations around the wavenumber k=1.00 may describe damped Turing patterns [31].

**Figure 3:**
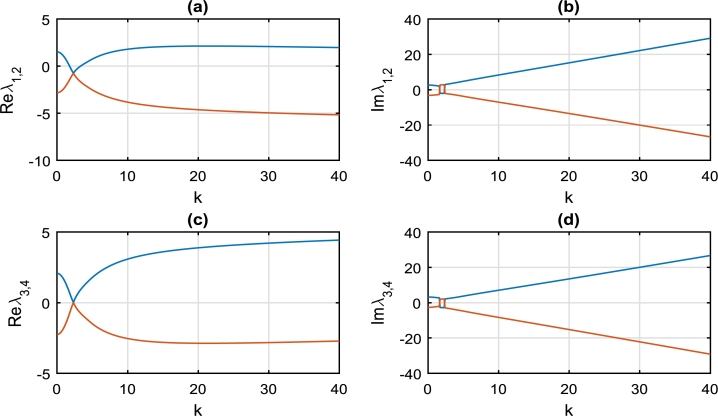
Variation of growth rate *λ* as a function of wavenumber *k*, dictated by solution (18a), (18b). This is evaluated at spatially-homogeneous state $\left(U_{2}^{⁎},\;V_{2}^{⁎})=(2.0,\;2.0\right)$ or $\left(U_{3}^{⁎},\;V_{3}^{⁎})=(-2.0,\;-2.0\right)$, with parameter values exactly as in Fig. 2.

We have equally considered the stability/instability map of perturbations around the spatial homogeneous state $\left(U_{2}^{⁎},\;V_{2}^{⁎}\right)$ or $\left(U_{3}^{⁎},\;V_{3}^{⁎}\right)$ as captured in Fig. 3. Just as in Fig. 2, we equally notice that there is instability of continuous waves in Fig. 3 for all values of k∈[0,40]; hence squeal noise is rife in the brake system. However around the critical perturbation wavenumber k=3.00 as highlighted in Fig. 3(a,c), the continuous wave becomes stable and the brake system is void of squeal noise.

## 4 Numerical investigations

It is important that we now consider a reduced time frame coordinate system [33] $\tau =T-v^{-1}x$, where *v* is the characteristic speed of the block masses. This transforms Eq. (10a), (10b) to the following coupled ordinary nonlinear differential equations

(20a) $$\frac{d^{2}U}{d\tau^{2}}=\gamma_{7}\frac{d^{2}U}{d\tau^{2}}-(U-\gamma_{2}V)(1+\gamma_{1}U^{2})+2\alpha \frac{dU}{d\tau },$$

(20b) $$\frac{d^{2}V}{d\tau^{2}}=-\gamma_{3}\frac{dV}{d\tau }+(\gamma_{4}U-\gamma_{5}V)(1+\gamma_{1}U^{2})-\gamma_{6}V,$$

where $\gamma_{7}={\left(al/v\right)}^{2}$. By further imposing the following transformations dU/dτ=Ψ,dV/dτ=Γ, Eq. (20a), (20b) reduces to a set of four coupled first-order nonlinear ordinary differential equations

(21a) $$\frac{dU}{d\tau }=\Psi ,$$

(21b) $$\frac{dV}{d\tau }=\Gamma ,$$

(21c) $$\frac{d\Psi }{d\tau }=\frac{1}{\left(1-\gamma_{7}\right)}[2\alpha \Psi -(U-\gamma_{2}V)(1+\gamma_{1}U^{2})],$$

(21d) $$\frac{d\Gamma }{d\tau }=-\gamma_{3}\Gamma +(\gamma_{4}U-\gamma_{5}V)(1+\gamma_{1}U^{2})-\gamma_{6}V.$$

We are now interested in the vibrational profiles of the block displacement *U*, pad position *V*, block velocity Ψ, and pad velocity Γ as a function of time *τ*, for $\gamma_{7}\neq 1$. Consequently, we now vary the values of the parameters: $\alpha ,\;\gamma_{1},\;\gamma_{2},\;\gamma_{3},\;\gamma_{4},\;\gamma_{5},\;\gamma_{6},\;\gamma_{7}$, and numerically simulate Eq. (21a), (21b), (21c), (21d) based on the fourth-order Runge-Kutta scheme with a time step of Δτ=0.01. This is because the linear stability analysis performed in the previous section which incorporate linearization around spatial equilibrium point, can not give a comprehensive account of the long term evolution of the brake system.

**Figure 4:**
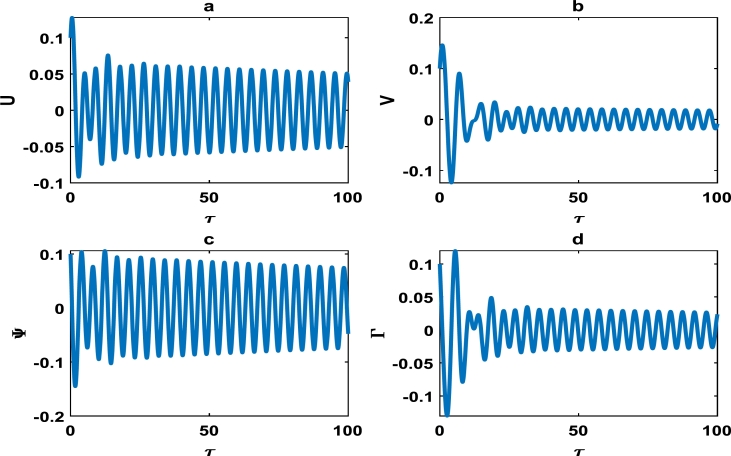
Evolution of block-pad brake dynamical system, based on the numerical integration of Eq. (21a), (21b), (21c), (21d). (a) Block displacement *U*, as a function of time *τ*. (b) Pad position *V*, as a function of time *τ*. (c) Block velocity Ψ, as a function of time *τ*. (d) Pad velocity Γ, as a function of time *τ*. This is for parameter values: *α* = 0.008, *γ*_1_ = 0.100, *γ*_2_ = 0.200, *γ*_3_ = 0.300, *γ*_4_ = 0.400, *γ*_5_ = 0.500, *γ*_6_ = 0.600, *γ*_7_ = 0.500.

**Figure 5:**
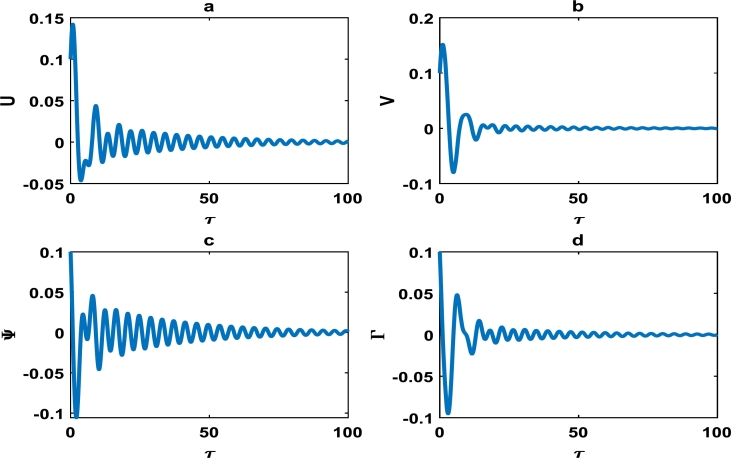
The description of vibrational profiles exactly as in Fig. 4, but for parameter values: *α* = 0.010, *γ*_1_ = 0.500, *γ*_2_ = 0.500, *γ*_3_ = 0.500, *γ*_4_ = 0.500, *γ*_5_ = 0.500, *γ*_6_ = 0.500, *γ*_7_ = 0.500.

The vibrations in Fig. 4 approximately captures harmonic oscillations of the displacement and velocity profiles of the block-pad brake system, as time increases. However for change of system parameters as in Fig. 5, there is exponential decrease in the vibrational dynamics of the block-pad brake system. Fig. 5 is a clear manifestation of stability in the dynamics of the brake system, because the oscillations rapidly die down as time increases. From a physical standpoint, such stability suggests that the automobile system may experience little or no brake squeal noise upon application of the brake force. Hence, there is more comfort in the automobile system. A keen observation of Fig. 4 shows that the value of the coefficient of the hardening spring is $\gamma_{1}=0.100$, while those of their counterparts in Fig. 5 is fixed at $\gamma_{1}=0.500$. This is a strong indication that we have to incorporate hardening springs during the fabrication of most automobile brake systems, so as to guarantee our comfort and safety.

**Figure 6:**
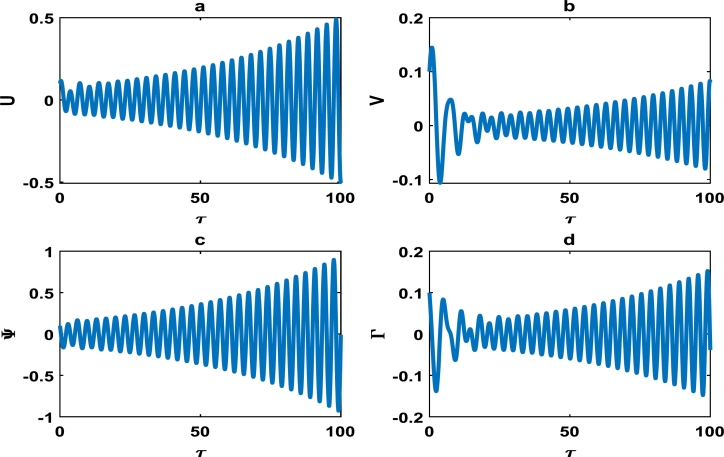
The description of vibrational profiles exactly as in Fig. 4, but for parameter values: *α* = 0.008, *γ*_1_ = 0.100, *γ*_2_ = 0.200, *γ*_3_ = 0.300, *γ*_4_ = 0.400, *γ*_5_ = 0.500, *γ*_6_ = 0.600, *γ*_7_ = 0.700.

**Figure 7:**
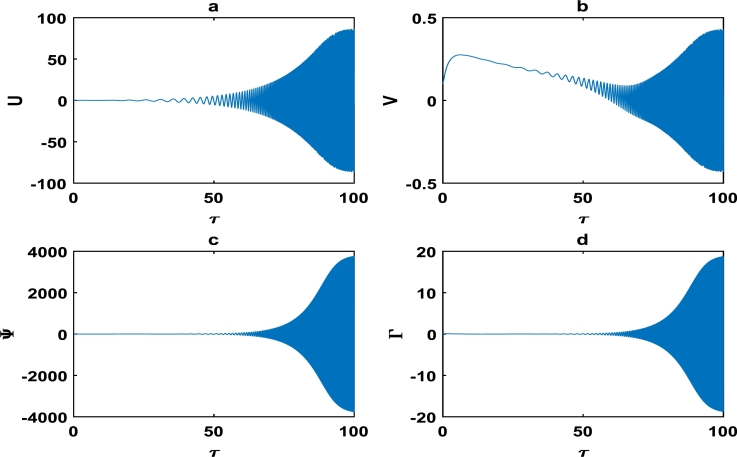
The description of vibrational profiles exactly as in Fig. 4, but for parameter values: *α* = 0.100, *γ*_1_ = 0.500, *γ*_2_ = 0.500, *γ*_3_ = 0.500, *γ*_4_ = 0.005, *γ*_5_ = 0.005, *γ*_6_ = 0.006, *γ*_7_ = 0.006.

**Figure 8:**
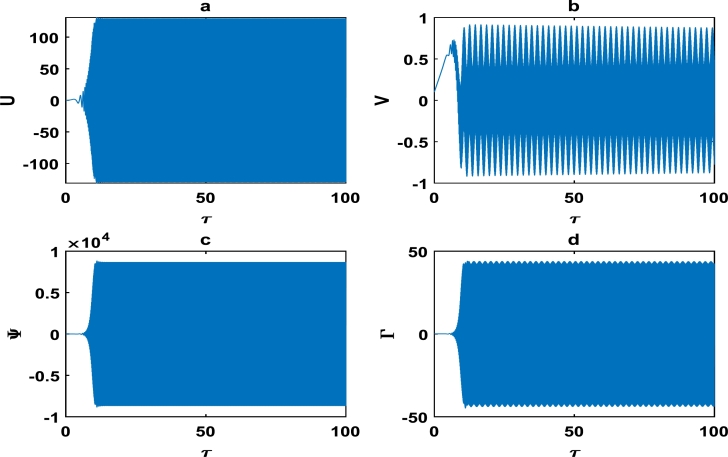
The description of vibrational profiles exactly as in Fig. 4, but for parameter values: *α* = 0.910, *γ*_1_ = 0.500, *γ*_2_ = 0.500, *γ*_3_ = 0.500, *γ*_4_ = 0.005, *γ*_5_ = 0.005, *γ*_6_ = 0.006, *γ*_7_ = 0.006.

**Figure 9:**
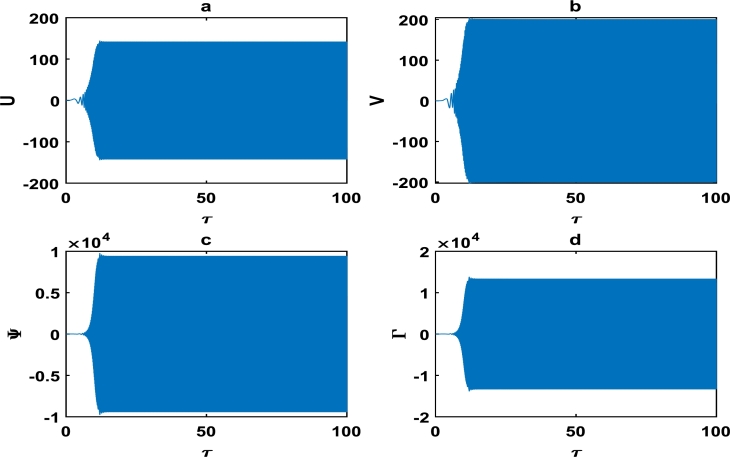
The description of vibrational profiles exactly as in Fig. 4, but for parameter values: *α* = 1.910, *γ*_1_ = 0.500, *γ*_2_ = 0.500, *γ*_3_ = 0.500, *γ*_4_ = 0.500, *γ*_5_ = 0.500, *γ*_6_ = 0.500, *γ*_7_ = −1.000.

**Figure 10:**
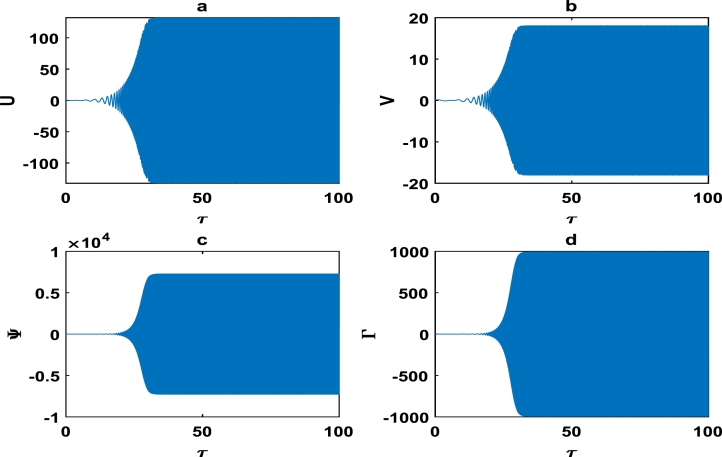
The description of vibrational profiles exactly as in Fig. 4, but for parameter values: *α* = 0.100, *γ*_1_ = 0.100, *γ*_2_ = 0.200, *γ*_3_ = 0.300, *γ*_4_ = 0.400, *γ*_5_ = 0.500, *γ*_6_ = 0.600, *γ*_7_ = 0.700.

**Figure 11:**
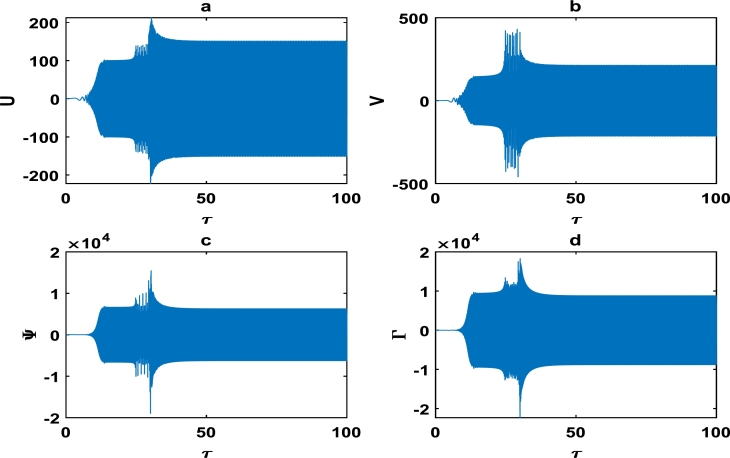
The description of vibrational profiles exactly as in Fig. 4, but for parameter values: *α* = 0.910, *γ*_1_ = 0.500, *γ*_2_ = 0.500, *γ*_3_ = 0.150, *γ*_4_ = 1.005, *γ*_5_ = 1.005, *γ*_6_ = 1.006, *γ*_7_ = 0.006.

The oscillations of the block-pad system exhibiting exponential growth as a function of time, are clearly captured in Fig. 6 as we further vary the parameters of the brake system. The exponential growth rate becomes more pronounce as from τ=75 in Fig. 7, hence Fig. 6 and Fig. 7 clearly highlight a high propensity of the brake system toward squeal noise. A careful observation of Fig. 7 reveals that the time frame of τ=100 is quite limited. This is because we noticed an exponential growth rate around τ=75, and can not tell with certainty whether the brake system continuous to stabilize or experience another instability beyond τ=100. This raises a pertinent issue of the unpredictability of the brake dynamics beyond instability, as underscored in the concluding remarks by Kinkaid et al. [11]. The Figure 8, Figure 9, Figure 10 depict a quasi-periodic oscillations of the brake system which signify stability, with the velocity amplitudes of the block-pad vibrations generally higher than their corresponding displacement amplitudes. A very interesting phenomenon is recorded in Fig. 11, where there is a non-linear transient oscillation around τ=30; followed by quasi-periodic vibrations. Such transient states which are characterized by highest amplitudes of vibrations, are crucial in predicting dangerous or favorable conditions of the brake system [32].

**Figure 12:**
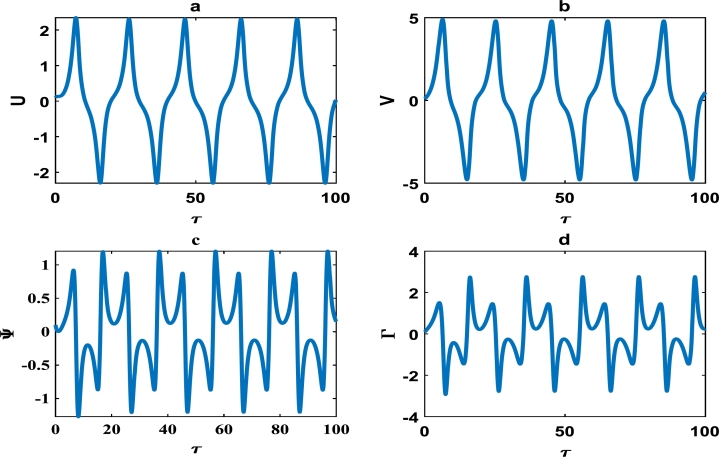
The description of vibrational profiles exactly as in Fig. 4, but for parameter values: *α* = −0.910, *γ*_1_ = 0.500, *γ*_2_ = 0.500, *γ*_3_ = −0.150, *γ*_4_ = 1.005, *γ*_5_ = 1.005, *γ*_6_ = −1.006, *γ*_7_ = −0.006.

Fig. 12(a,b) depicts the stick-slip motion of the block-pad system, with approximately uniform amplitude and period. The corresponding velocity profiles of the stick-slip block-pad motion are captured in Fig. 12(c,d).

**Figure 13:**
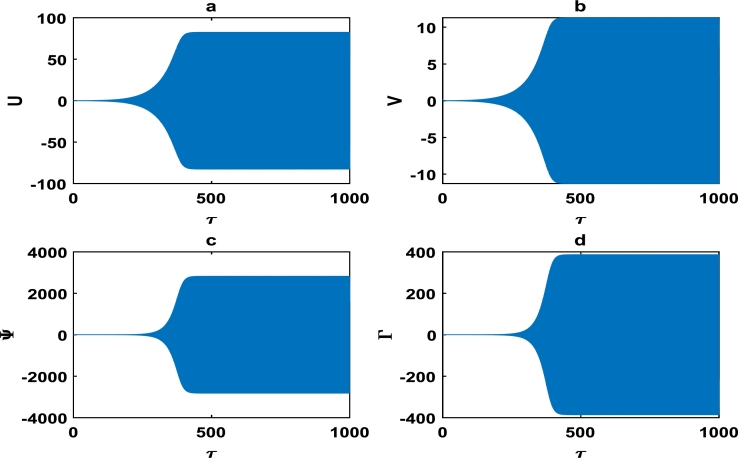
The description of vibrational profiles exactly as in Fig. 4, but for parameter values: *α* = 0.008, *γ*_1_ = 0.100, *γ*_2_ = 0.200, *γ*_3_ = 0.300, *γ*_4_ = 0.400, *γ*_5_ = 0.500, *γ*_6_ = 0.600, *γ*_7_ = 0.700.

**Figure 14:**
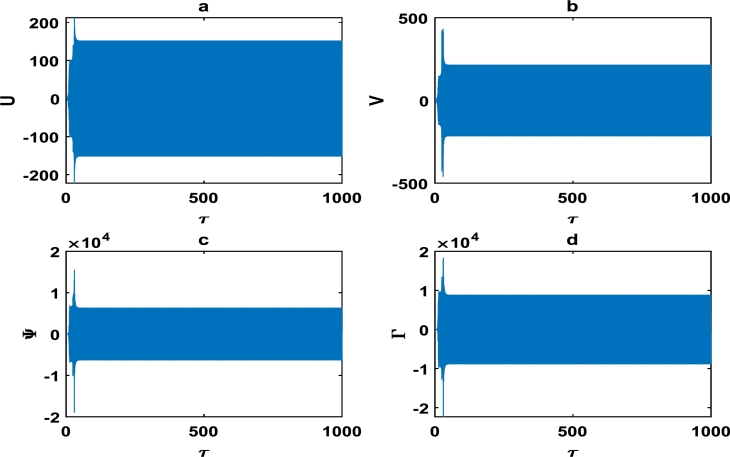
The description of vibrational profiles exactly as in Fig. 4, but for parameter values: *α* = 0.910, *γ*_1_ = 0.500, *γ*_2_ = 0.500, *γ*_3_ = 0.150, *γ*_4_ = 1.005, *γ*_5_ = 1.005, *γ*_6_ = 1.006, *γ*_7_ = 0.006.

We further extend the time frame of vibrations up to 1000, in order to obtain the long term structural profiles of the block-pad dynamics given in Fig. 13 and Fig. 14. We observe in Fig. 13 that there is an exponential increase in the block-pad vibration amplitude within time frame τ∈[200,400], followed by constant amplitude oscillations. Just as in Fig. 11, the transient non-linear oscillation phenomenon is identified in the early stages of wave profile in Fig. 14. The block-pad oscillation of the brake system in Fig. 13 and Fig. 14, clearly captures long term stability that is void of brake squeal noise.

## 5 Discussion and conclusion

This scientific investigation hinges on proposing a basic theoretical brake system and exploiting the theory of nonlinear dynamical systems, in order to characterize the stability of small amplitude vibrations. We understand the difficulties inherent in the automobile industry, to clearly solve the elusive problem of disc brake squeal noise. We believe that the innovative exploitation of nonlinear brake models, will offer new perspectives with robust solutions to disc brake squealing. The friction coefficient *α* was systematically varied, and various profile curves shown from Fig. 4 to Fig. 14 were obtained. Our results clearly suggest that the *α*−coefficient remains an intrinsic parameter in the brake dynamics. However, we can not state a precise critical value of *α* beyond/below which brake squeal may occur. This is because our proposed brake model is characterized by many other parameters, which collectively determine the holistic dynamics of our system.

Friction-induced oscillations developed in brake mechanisms are inextricably linked to the instability of the system as a whole. For example, unstable modes which may be a precursor of squealing in a disc brake system were derived via complex eigenvalue analysis [34]. Massi et al. used the finite element modal numerical analysis on a brake system to predict the onset of squeal, while a specific finite element programme for nonlinear dynamical analysis was fully incorporated to demonstrate the squeal phenomena in the time domain [8]. The stability analysis around an equilibrium point can likely be used as an initial step of predicting intrinsic vibrations in the disc brake system, which may trigger squealing [32]. Consequently, the linear stability analysis performed around an equilibrium point in Section 3 can only be used to characterize the onset of instability in the brake system. A more comprehensive analysis of our brake system which depicts nonlinear transient states among other interesting oscillations; were carefully handled in Section 4. The big task now is to properly examine whether there is consistency between the linear stability analysis performed in Section 3 and the numerical simulations carried out in Section 4. A careful observation of the plots in Fig. 2, Fig. 3 and Fig. 5 shows that the friction parameter *α* is fixed at 0.010, while the minimum value of all other parameters (i.e. $\gamma_{i},\;i=1,2,3,4,5,6,7$) is 0.500. Fig. 5 clearly captures a damping pattern of the brake dynamics, which signify stability. This was clearly predicted in our linear stability analysis for wavenumber *k* around 1.00 and spatial homogeneous state $\left(U^{⁎},\;V^{⁎})=(0,\;0\right)$. Fig. 4 equally manifest stable patterns, even though it is not as pronounce as in Fig. 5. On the other hand, all the curves from Fig. 6 to Fig. 14; clearly captures the onset of exponential growth. This is equally in conformity with the linear stability analysis, that predicted unstable patterns for most of the parameter values. Our theoretical results therefore suggest a holistic correlation between the linear stability and numerical analyses to a good extent.

We have considered the dynamics of a brake system governed by modified Burridge-Knopoff-Pad model, where we carried out a linear stability and numerical analyses of the system. It is quite useful to stress that within the context of designing mechanical brake systems, our proposed model is quite innovative. However, from the perspectives of modeling mechanical systems in general; the use of nonlinear springs whose spring constant is dependent on the block displacements have already been highlighted in a study carried out by Nfor et al. on earthquake dynamics [22]. The basic theoretical modified Burridge-Knopoff-Pad model of disc brake system, thus furnishes us with valid information on the generation of brake squeal noise and a plethora of self excited non-linear vibrations. Notably is the emergence of transient states, with the highest possible amplitude of oscillations.

The design of more robust brake systems in which transient states are accurately predicted and squeal noise greatly minimized, remains a great challenge to mechanical engineers. This is exacerbated by the fact that many questions in the domain of friction-induced vibrations are poorly understood, hence putting the safety and reliability of most complex automobile brake systems questionable.

## Funding

The authors did not receive support from any organization for the submitted work.

## CRediT authorship contribution statement

**Oma Nfor Nkeh:** Writing – original draft, Project administration, Investigation, Conceptualization. **Akoni Brikly Njinabo:** Software. **Waindim Yisa Tufoin Albert:** Writing – original draft. **Kenfack Djifack Hunnel:** Writing – review & editing.

## Declaration of Competing Interest

The authors declare that they have no known competing financial interests or personal relationships that could have appeared to influence the work reported in this paper.

## Data Availability

The data used to support the findings of this study are included in this article.
